# Selective CD28 blockade impacts T cell differentiation during homeostatic reconstitution following lymphodepletion

**DOI:** 10.3389/fimmu.2022.1081163

**Published:** 2023-01-24

**Authors:** Jakob G. Habib, Danya Liu, Rebecca M. Crepeau, Maylene E. Wagener, Mandy L. Ford

**Affiliations:** Emory Transplant Center, Department of Surgery, Emory University School of Medicine, Atlanta, GA, United States

**Keywords:** costimulation blockade, T cell depletion, lymphodepletion, homeostatic reconstitution, transplantation, alloimmunity

## Abstract

**Introduction:**

Costimulation blockade targeting the CD28 pathway provides improved long-term renal allograft survival compared to calcineurin inhibitors but may be limited as CTLA-4-Ig (abatacept, belatacept) blocks both CD28 costimulation and CTLA-4 coinhibition. Directly targeting CD28 while leaving CTLA-4 intact may provide a mechanistic advantage. Fc-silent non-crosslinking CD28 antagonizing domain antibodies (dAb) are currently in clinical trials for renal transplantation. Given the current standard of care in renal transplantation at most US centers, it is likely that lymphodepletion via thymoglobulin induction therapy could be used in patients treated with CD28 antagonists. Thus, we investigated the impact of T cell depletion (TCD) on T cell phenotype following homeostatic reconstitution in a murine model of skin transplantation treated with anti-CD28dAb.

**Methods:**

Skin from BALB/cJ donors was grafted onto C56BL/6 recipients which were treated with or without 0.2mg anti-CD4 and 10μg anti-CD8 one day prior to transplant and with or without 100μg anti-CD28dAb on days 0, 2, 4, 6, and weekly thereafter. Mice were euthanized six weeks post-transplant and lymphoid cells were analyzed by flow cytometry.

**Results:**

Anti-CD28dAb reversed lymphopenia-induced differentiation of memory CD4+ T cells in the spleen and lymph node compared to TCD alone. Mice treated with TCD+anti-CD28dAb exhibited significantly improved skin graft survival compared to anti-CD28dAb alone, which was also improved compared to no treatment. In addition, the expression of CD69 was reduced on CD4+ and CD8+ T cells in the spleen and lymph node from mice that received TCD+anti-CD28dAb compared to TCD alone. While a reduced frequency of CD4+FoxP3+ T cells was observed in anti-CD28dAb treated mice relative to untreated controls, this was balanced by an increased frequency of CD8+Foxp3+ T cells that was observed in the blood and kidney of mice given TCD+anti-CD28dAb compared to TCD alone.

**Discussion:**

These data demonstrate that CD28 signaling impacts the differentiation of both CD4+ and CD8+ T cells during homeostatic reconstitution following lymphodepletion, resulting in a shift towards fewer activated memory T cells and more CD8+FoxP3+ T cells, a profile that may underpin the observed prolongation in allograft survival.

## Introduction

Belatacept is the first costimulation blockade therapy approved for use in the clinic for renal transplantation (1, 2). It is comprised of a CTLA-4-Ig fusion protein that binds to CD80 and CD86 on antigen presenting cells (APCs), preventing CD28 costimulation on T cells. Costimulation blockade with belatacept results in less nephrotoxicity, better kidney function, and significantly improved long-term graft survival compared to calcineurin inhibitors (3). However, treatment with belatacept is also associated with increased rates and severity of acute rejection (4). One potential cause for this may be due to the lack of CTLA-4 coinhibition, as CTLA-4 on T cells also binds to CD80 and CD86 on APCs but is blocked by belatacept. CTLA-4 functions to suppress T cell responses in both a cell intrinsic and extrinsic manner and is differentially expressed on different T cell subsets (5–8). CTLA-4 suppresses alloreactive responses in a cell-intrinsic manner on CD8^+^ memory T cells (9) and Th17 cells, both of which have been shown to drive costimulation blockade resistant rejection (10–13). On regulatory T cells (Tregs), CTLA-4 provides non-redundant immunosuppressive signals in a cell-extrinsic manner (14). Treg-specific CTLA-4 deficiency in mice leads to spontaneous development of systemic lymphoproliferation and fatal T cell-mediated autoimmune disease (15). Therefore, selectively blocking CD28 while leaving CTLA-4 signaling intact may prove beneficial in a transplant setting.

Recently, selective CD28 blockers such as the anti-CD28 domain antibody (dAb) lulizumab (BMS-931699) and anti-CD28 Fab’ FR104 (VEL-101) have been developed (16). These selective CD28 blockers have been shown to be equally able to inhibit CD80-elicited T cell proliferation and five times more potent at inhibiting CD86-elicited T cell proliferation as compared to belatacept (17). Importantly, Fc-silent non-crosslinking reagents do not elicit cytokine release, unlike the earlier Fc-containing anti-CD28 TGN1412 (18, 19). These reagents may also be more potent against alloimmune responses than belatacept, yet are comparable to belatacept in terms of their impact on CD8^+^ T cell protective immunity in a murine EBV homolog model (20). Moreover, selective CD28 blockade attenuates CD8^+^ memory T cell effector function in a CTLA-4-dependent manner during murine skin transplantation (21). In a model of murine cardiac allotransplantation, Zhang et al. (22) demonstrated that selective CD28 blockade attenuated acute and chronic graft rejection which was dependent on the preservation of CTLA-4 signaling. Using a single chain monovalent non-activating reagent to block CD28 signaling (sc28AT), Poirier et al. (23) showed that in nonhuman primate models of renal and cardiac transplantation, selective CD28 blockade synergized with calcineurin inhibitors to improve the frequency and function of Tregs and prolong graft survival. Further, Zhang et al. (24) demonstrated improved cardiac allograft survival and reduced T cell activation in nonhuman primates treated with sc28AT induction followed by anti-CD154 administration. Additionally, FR-104 was shown to prevent acute rejection in nonhuman primate renal allografts, and to promote the accumulation of Tregs in the blood and graft without the need for steroids (16). This reagent has also been shown to be safe for administration in humans (25).

Future clinical trials investigating the efficacy of selective CD28 blockers during renal transplantation will likely include induction therapy *via* administration of anti-thymocyte globulin (ATG) (26, 27). The impact of selective CD28 blockade on T cell depletion (TCD) and subsequent homeostatic reconstitution of T cells is not known. Prlic et al. (28) found that CD28 signaling was dispensable for the bulk reconstitution of CD4^+^ and CD8^+^ T cells as measured by CFSE proliferation. While informative, this study used CD28-knockout mice as opposed to the selective anti-CD28dAb that is now available. In addition, the impact on specific T cell subsets beyond bulk CD4^+^ and CD8^+^ T cell populations, such as regulatory T cells and memory T cell subsets, was not assessed. Thus, here we investigated the impact of selective CD28 blockade on T cell phenotype following TCD and homeostatic reconstitution in a murine model of skin transplantation. We demonstrated that CD28 signaling impacts the differentiation of both CD4^+^ and CD8^+^ T cells during homeostatic reconstitution following lymphodepletion, resulting in a shift towards fewer activated memory T cells in lymphoid organs and more CD8^+^Foxp3^+^ T cells in the blood and kidney. This favorable immune profile is accompanied by improved skin graft survival and suggests that selective CD28 blockers could be efficacious at controlling allograft rejection in patients in the context of T cell depletion.

## Materials and methods

### Mice

C57BL/6J and BALB/cJ mice were obtained from the National Cancer Institute (Frederick, MD). All animals were maintained in accordance with Emory University Institutional Animal Care and Use Committee guidelines. All animals were housed in specific pathogen-free animal facilities at Emory University.

### Skin transplantation and *in vivo* co-stimulatory molecule blockade

Full-thickness BALB/cJ tail and ear skin grafts were transplanted onto the dorsal thorax of recipient C57BL/6J mice and secured with adhesive bandages as previously described (11). Where indicated, mice were injected with 100µg anti-CD28 dAb (Bristol-Myers Squibb) on days 0, 2, 4, 6, and weekly thereafter until the mice were sacrificed on day 42. For T cell depletion experimental groups, 0.2mg of anti-CD4 (clone GK1.5) and 10μg of anti-CD8 (clone 2.43; both from BioXcell) were administered one day prior to skin transplantation.

### Intravascular labeling and tissue processing

Blood was collected weekly to assess T cell phenotype and absolute count. On day 42 prior to organ harvest, mice were intravenously injected with 1.5µg of anti-CD4-BV650 and anti-CD8a-BV650 antibody 2 minutes before euthanasia to label circulating blood cells. The spleen, axillary lymph nodes, bone marrow, kidneys, liver, lungs, and blood were then procured. Kidney and lung samples were chopped and digested for 30 minutes at 37˚C with 2mg/ml Collagenase (type 4, Sigma-Aldrich) and 50µg/ml DNAse (ThermoFisher) in HBSS. Digested lungs were then homogenized, filtered, and washed in FACS buffer (PBS with 2% FBS). Livers were homogenized manually, filtered through 40μm strainers, and spun lightly at 300rpm to pellet the hepatocytes. The liver supernatant and digested kidneys were resuspended in a 40% Percoll solution, overlaid on 70% Percoll, and spun at 2000rpm for 20 minutes with the brake off. The buffy coats were isolated and washed in FACS buffer. Bone marrow from tibia and fibula were flushed with a 21-gauge needle using PBS and homogenized into single cell suspensions. Spleens and lymph nodes were processed into single cell suspensions, and blood, spleen, and bone marrow were lysed with Fixative-Free Lysing Solution following manufacturer’s instructions (Invitrogen). Each tissue was then washed in FACS buffer and stained with antibodies for flow cytometry.

### Flow cytometry

For phenotypic analysis, cells were surface-stained with: TIGIT-BV421, CD103-BV605, CD69-BV711, PD-1-BV786, CD28-FITC, CD49d-PE, CD122-PE-Cy7, DNAM-1 (CD226)-APC, CX3CR1-Alexa700, CD27-APC-Cy7, CTLA-4 (CD152)-BV421, TIM3-BV605, OX40-BV711, CD8-BV786, 2B4 (CD244)-FITC, CD62L-PerCP-Cy5.5, ICOS-PE-Dazzle, CD43-PE-Cy7, CD44-Alexa700, CD25-APC-Cy7 (BioLegend), CD8-PacOrange, CD127-APC (ThermoFisher), CD4-BUV395, CXCR3-BV510 (BD). For Fc staining, cells were incubated with biotinylated CD16/CD32 (2.4G2, BD Biosciences), washed twice, and stained with streptavidin-PerCP-Cy5.5 (BioLegend). For transcription factor staining, cells were permeabilized using a FoxP3/transcription factor kit (Invitrogen) and stained with FoxP3-PE (ThermoFisher). Absolute numbers were calculated using CountBright Absolute Counting Beads according to the manufacturer’s instructions (ThermoFisher). Samples were analyzed on an LSRFortessa flow cytometer (BD). Data were analyzed using FlowJo software (Tree Star).

### Statistical analysis

Two-way ANOVA with multiple comparisons was performed when comparing multiple groups. Survival data were plotted on Kaplan-Meier curves, and a log-rank (Mantel-Cox) test was performed. All analyses were done using GraphPad Prism version 9.4.1 for Mac, GraphPad Software, San Diego, California USA. In all legends and figures, mean ± SD is shown, and *p<0.05, **p<0.01, ***p<0.001, ****p<0.0001.

## Results

### Selective CD28 blockade reverses lymphopenia-induced differentiation of memory CD4^+^ T cells in the spleen and lymph node

**Figure 1:**
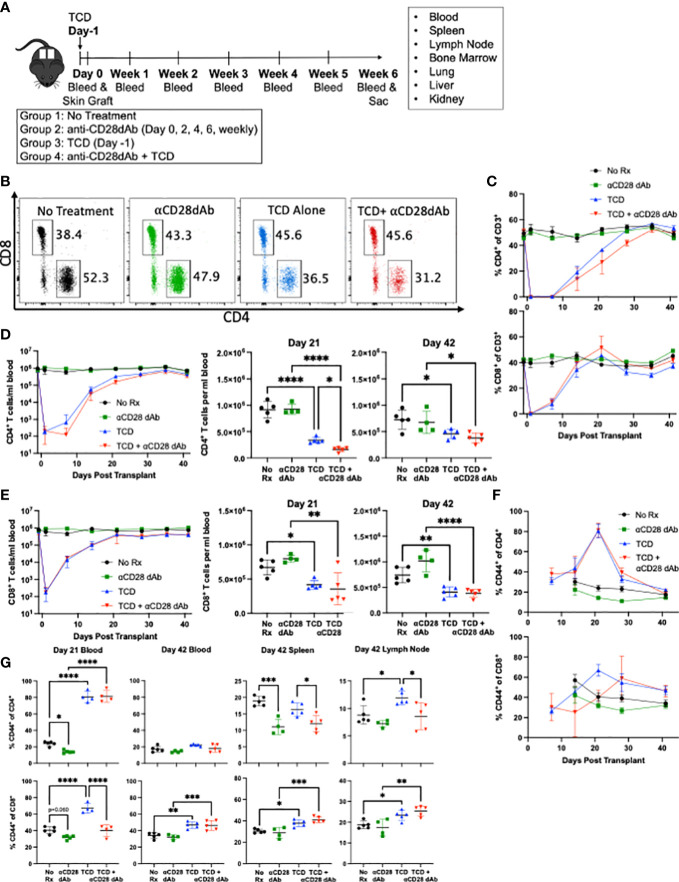
Selective CD28 blockade reverses lymphopenia-induced differentiation of memory CD4^+^ T cells in the lymph node. **(A)** Fully MHC-mismatched skin from BALB/c donors were grafted onto C57BL/6 recipients. Recipients were treated with or without 0.2mg anti-CD4 and 10μg anti-CD8 one day prior to transplant and with or without 100μg anti-CD28dAb on days 0, 2, 4, 6, and weekly thereafter. Mice were euthanized six weeks post-transplant and lymphoid cells from the blood, lymph node, spleen, bone marrow, kidney, liver, and lung were analyzed by flow cytometry. Immediately prior to euthanasia fluorescently labeled anti-CD4 and anti-CD8 antibody were administered IV to label circulating T cells. **(B)** Representative flow cytometry plots of CD4^+^ and CD8^+^ T cells on day 21 in the blood. **(C)** Frequency of CD4^+^ and CD8^+^ T cells in the blood over time. **(D)** Left, absolute count of CD4^+^ T cells per mL of blood over time. Right, absolute count of CD4^+^ T cells at day 21 and day 42. **(E)** Left, absolute count of CD8^+^ T cells per mL of blood over time. Right, absolute count of CD8^+^ T cells at day 21 and Day 42. **(F)** Proportion of CD44 expressing CD4^+^ and CD8^+^ T cells in the blood over time. **(G)** Proportion of CD44 expressing CD4^+^ and CD8^+^ T cells in the blood at day 21 and in the blood, spleen, and lymph node at day 42. Experiment shown is representative of 2 independent experiments with a total of 8-10 mice per group. *p < 0.05, **p < 0.01, ***p < 0.001, ****P < 0.0001 by one way ANOVA correcting for multiple comparisons.

To investigate the impact of selective CD28 blockade on immune reconstitution following T cell depletion (TCD) in the setting of transplantation, C57BL/6 mice were randomized to receive either vehicle controls or anti-CD4 and anti-CD8 depleting antibody one day prior to receiving a fully MHC-mismatched BALB/c skin graft. Following transplantation, mice in each group were further randomized to receive either no further treatment or anti-CD28dAb (**Figure 1A**). Flow cytometric analysis of peripheral blood was used to monitor T cell depletion on the day of transplantation and blood was drawn weekly to monitor the kinetics of T cell reconstitution (**Figures 1A–G**). Results indicated that across the majority of timepoints, the frequencies of reconstituted CD4^+^ and CD8^+^ T cells among CD3^+^ cells were largely unaffected by CD28 blockade (**Figure 1C**). The exception to this was the fact that the absolute count of CD4^+^ T cells was reduced on day 21 post-transplantation in the TCD+anti-CD28dAb group as compared to TCD alone (p<0.05), but was no different by day 42, indicating that CD4^+^ T cell reconstitution is delayed in the absence of CD28 signaling but ultimately not inhibited (**Figure 1D**). No differences in the reconstitution kinetics of CD8^+^ T cells between the TCD alone and the TCD+anti-CD28dAb groups were observed (**Figure 1E**). Interestingly, the absolute count of both CD4^+^ and CD8^+^ T cells remained lower in the TCD groups compared to non-depleted groups, regardless of CD28 blockade status (**Figures 1D, E**).

While no differences in the magnitude of the CD4^+^ (**Figure 1D**) and CD8^+^ T cell compartments (**Figure 1E**) were observed following T cell reconstitution in the presence or absence of selective CD28 blockade, we next sought to determine if selective CD28 blockade impacted memory T cell phenotype under these conditions. Results indicated that at the earlier timepoint (day 21), TCD resulted in increased frequencies of CD44^+^ effector/memory T cells within both CD4^+^ and CD8^+^ T cell compartments (p<0.0001 for both) (**Figure 1G**). At day 21 for CD8^+^ T cells, selective CD28 blockade of TCD-treated animals resulted in a significant reduction in the frequency of CD44^+^ effector/memory T cells (p<0.0001). This finding was also observed within the CD4^+^ T cell compartment but at the later timepoint (day 42), in that selective CD28 blockade of TCD-treated animals resulted in a significant reduction in the frequency of CD44^+^ effector/memory CD4^+^ T cells in both the spleen (p=0.021) and lymph node (p=0.042) (**Figure 1G**). Overall, these results indicate that T cell depletion results in an increased frequency of effector/memory CD44^+^ T cells within both the CD4^+^ and CD8^+^ T cell compartments, an effect which is mitigated by the addition of selective CD28 blockade at d21 in the blood for CD8^+^ T cells and at d42 in secondary lymphoid organs for CD4^+^ T cells.

### Selective CD28 blockade during T cell lymphodepletion and reconstitution improves skin graft survival and reduces expression of CD4^+^ T cell activation and senescence markers

**Figure 2:**
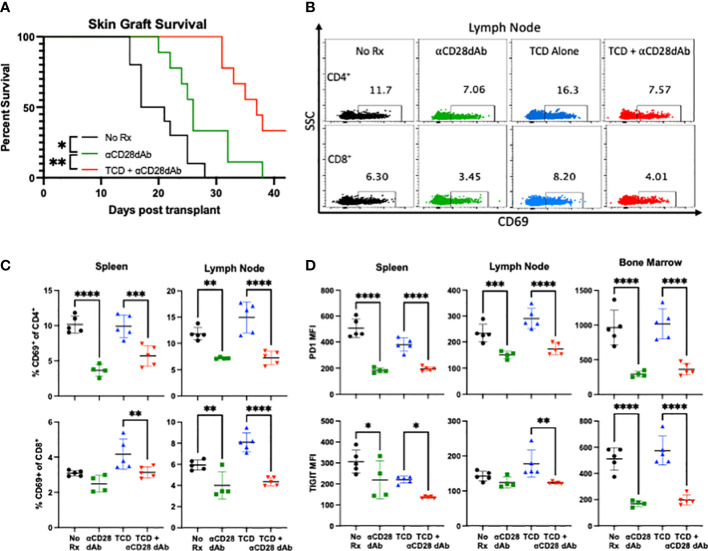
Selective CD28 blockade during T cell lymphodepletion and reconstitution improves skin graft survival and reduces expression of CD4^+^ T cell activation and senescence markers. **(A)** Median survival time of skin grafts was 19 days with no treatment (No Rx), 26 days for CD28dAb alone, and 37 days for CD28dAb+TCD. *p<0.05, **p < 0.01 by Mantel-Cox log-rank test. **(B)** Representative flow cytometric data depicting the frequency of CD69^+^CD4^+^ and CD69^+^CD8^+^ T cells in the lymph node at day 42. **(C)** Frequency of CD69^+^ CD4^+^ and CD8^+^ T cells in the spleen and lymph node at day 42. **(D)** MFI of PD-1 and TIGIT on CD4^+^ T cells in the spleen, lymph node, and bone marrow at day 42. Experiment shown is representative of 2 independent experiments with a total of 8-10 mice per group. *p < 0.05, **p < 0.01, ***p < 0.001, ****p < 0.0001 by one way ANOVA correcting for multiple comparisons.

To assess the impact of TCD on anti-CD28dab mediated graft survival, full-thickness MHC-mismatched skin grafts were performed as described above and graft survival was monitored for six weeks. While skin grafts in groups treated solely with anti-CD28dAb exhibited increased survival relative to untreated animals (median survival time [MST] 26 days vs. 19 days, p=0.016), the combination of TCD+anti-CD28dAb further improved skin graft survival compared to no treatment (MST 37 days, p=0.006, **Figure 2A**). To investigate the combined impact of anti-CD28dAb and TCD on immune activation, the proportion of CD69^+^ T cells among CD4^+^ and CD8^+^ T cell populations in the spleen and lymph node was examined (**Figures 2B, C**). Results demonstrated a reduced frequency of CD69-expressing CD4^+^ T cells in the spleen and lymph node in animals treated with anti-CD28dAb versus animals that did not receive the blockade, both in the presence and absence of TCD (**Figure 2C**). A similar trend was observed within the CD8^+^ T cell compartment, though in the spleen no differences in CD69-expressing CD8^+^ T cells were observed between the no treatment group and the anti-CD28dAb treated group (**Figure 2C**).

Next, to investigate the impact of TCD and selective CD28 blockade on T cell senescence, the expression of the coinhibitory receptors PD-1 and TIGIT were investigated (**Figure 2D**). In general, anti-CD28dAb treatment reduced the expression of both PD-1 and TIGIT on CD4^+^ T cells, both in the presence and absence of T cell depletion (**Figure 2D**). No difference was observed in the expression of PD-1 or TIGIT on CD8^+^ T cells between any of the groups (data not shown). Taken together, these results suggest that TCD combined with CD28 blockade improves skin graft survival, and that anti-CD28dAb reduces CD4^+^ T cell activation and expression of the coinhibitory receptors PD-1 and TIGIT.

### Selective CD28 blockade reduces the frequency of CD4^+^ and CD8^+^ T_RM_ in the kidney in the absence of T cell lymphopenia-induced reconstitution

**Figure 3:**
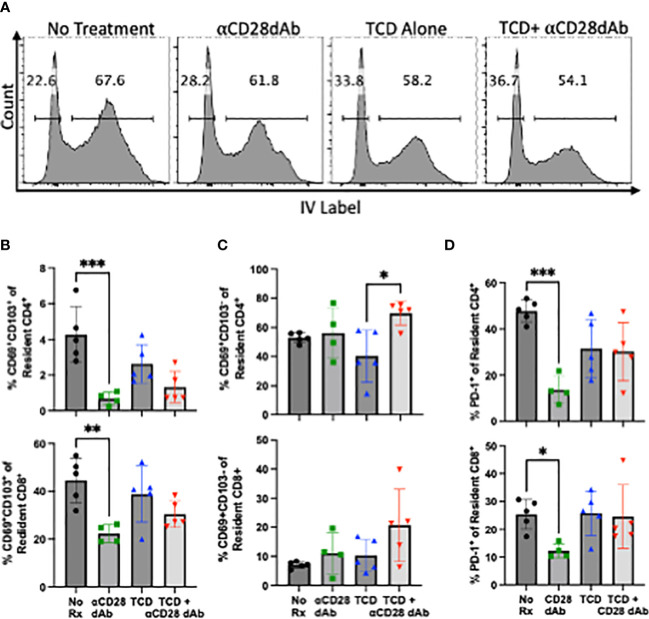
Selective CD28 blockade reduces the frequency of CD4^+^ and CD8^+^ T_RM_ in the kidney but not during T cell lymphopenia-induced reconstitution. **(A)** Flow cytometric gating strategy to identify tissue resident T cells in the kidney. **(B)** Frequency of tissue resident CD69^+^CD103^+^ CD4^+^ and CD8^+^ T cells in the kidney at day 42. **(C)** Frequency of tissue resident CD69^+^CD103^-^ CD4^+^ and CD8^+^ T cells in the kidney at day 42. **(D)** Frequency of PD-1^+^ resident CD4^+^ and CD8^+^ T cells in the kidney day 42. N=3-5 mice per group. *p < 0.05, **p < 0.01, ***p < 0.001, by one way ANOVA correcting for multiple comparisons.

Given the above results which demonstrate the impact of selective CD28 blockade on the homeostatic reconstitution of circulating memory CD4^+^ T cells following TCD, the impact of selective CD28 blockade on the tissue resident memory T cell (T_RM_) compartment was subsequently investigated, as this subset has been recently identified as a mediator of allograft rejection (29). To distinguish between circulating and tissue resident T cells, 1.5µg of fluorescently-labeled anti-CD4 and anti-CD8 were injected into the tail vein of each animal 2-3 minutes prior to euthanasia. Upon flow cytometric analysis of homogenized kidney, circulating CD4^+^ and CD8^+^ T cells stain positively for this IV label, while tissue resident cells are negative (**Figure 3A**).

Tissue resident memory T cells can be identified by their expression of CD69 with additional subsets, particularly CD8^+^ T cells, co-expressing CD103 (30–36). In contrast to the clear impact of TCD and homeostatic reconstitution on the frequencies of circulating memory T cells (**Figure 1G**), TCD did not significantly impact the frequency of CD69^+^CD103^+^ T_RM_ within either the CD4^+^ or CD8^+^ T cell compartment in the kidney. Administration of anti-CD28dAb in the absence of TCD resulted in a significant reduction in the frequency of CD69^+^CD103^+^ CD4^+^ (p=0.001) and CD8^+^ (p=0.005) T cells in the kidney (**Figure 3B**). In groups that received anti-CD28dAb in the setting of T cell homeostatic reconstitution, there was no difference in the frequency of CD69^+^CD103^+^ among CD4^+^ or CD8^+^ T cells compared to groups that received TCD alone (**Figure 3B**). As CD69^+^CD103^-^ T_RM_ have been described, particularly among CD4^+^ T cells (37–40), the frequency of CD69^+^CD103^-^ T_RM_ was further investigated. In the kidney, the frequency of CD4^+^CD69^+^CD103^-^ T cells was elevated (p=0.0103) in the group that received anti-CD28dAb in the setting of T cell homeostatic reconstitution compared to the group that received TCD alone (**Figure 3C**). There were no differences in the frequencies of CD8^+^CD69^+^CD103^-^ T cells among any of the groups in the kidney (**Figure 3C**). Moreover, the frequency of PD-1 expressing CD4^+^ and CD8^+^ T cells in the kidney (p=0.0002, p=0.047) were reduced in the groups that received anti-CD28dAb compared to the no treatment groups (**Figure 3D**).

### Selective CD28 blockade reduces the frequency of FoxP3^+^ CD4^+^ T cells but increases the frequency of FoxP3^+^ CD8^+^ T cells following T cell homeostatic reconstitution

**Figure 4:**
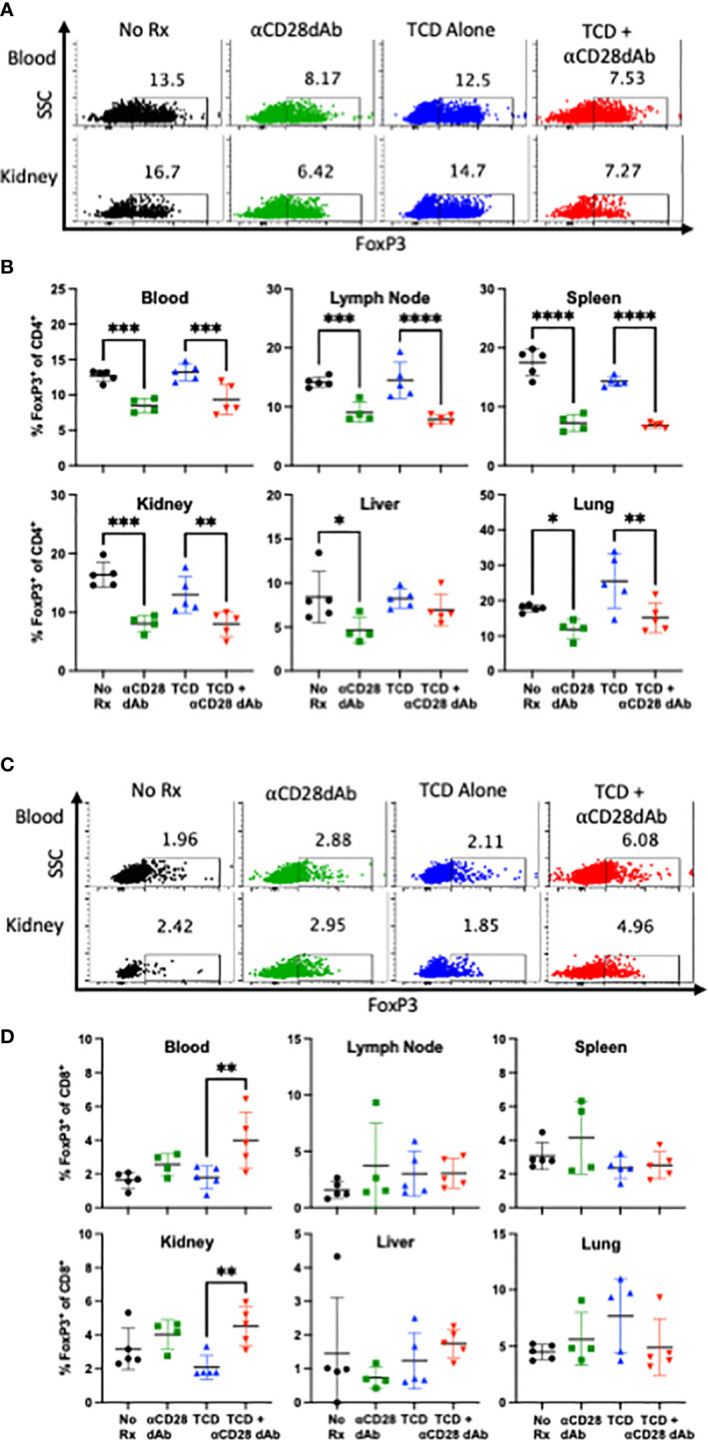
Selective CD28 blockade reduces the frequency of FoxP3^+^ CD4^+^ T cells but increases the frequency of FoxP3^+^ CD8^+^ T cells following T cell homeostatic reconstitution. **(A)** Representative flow cytometric data depicting the frequency of FoxP3^+^CD4^+^ T cells in the blood and kidney at day 42 **(B)** Frequency of FoxP3^+^CD4^+^ T cells in the blood, lymph node, spleen, kidney, liver, and lung at day 42. **(C)** Representative flow cytometric data depicting the frequency of FoxP3^+^CD8^+^ T cells in the blood and kidney at day 42. **(C)** Frequency of FoxP3^+^CD8^+^ T cells in the blood, lymph node, spleen, kidney, liver, and lung at day 42. Experiment shown is representative of 2 independent experiments with a total of 8-10 mice per group. *p < 0.05, **p < 0.01, ***p < 0.001, ****p < 0.0001, by one way ANOVA correcting for multiple comparisons.

Seminal studies have shown that CD4^+^FoxP3^+^ Tregs require CD28 signaling for homeostatic maintenance and survival (41). As such, we sought to determine the impact of selective CD28 blockade on CD4^+^FoxP3^+^ Tregs during homeostatic reconstitution following transplantation. Animals treated with anti-CD28dAb exhibited reduced frequencies of FoxP3^+^ cells among CD4^+^ T cells in the blood, lymph node, spleen, kidney, liver, and lung (**Figures 4A, B**), confirming the requirement of CD28 signaling for Treg maintenance.

There has been growing interest in CD8^+^ FoxP3^+^ T cells as potential mediators of immunological tolerance (42–45). Thus, the impact of selective CD28 blockade on this subset was investigated in the setting of transplantation and immune reconstitution. It was found that in contrast to the effect of selective CD28 blockade on CD4^+^Foxp3^+^ Tregs, animals treated with anti-CD28dAb in the setting of TCD actually exhibited an increased frequency of CD8^+^ Foxp3^+^ T cells in the blood and kidney (**Figures 4C, D**).

### CD8^+^ FoxP3^+^T cells exhibit distinct cell surface expression profiles compared to CD4^+^FoxP3^+^ T cells in the blood and kidney

**Figure 5:**
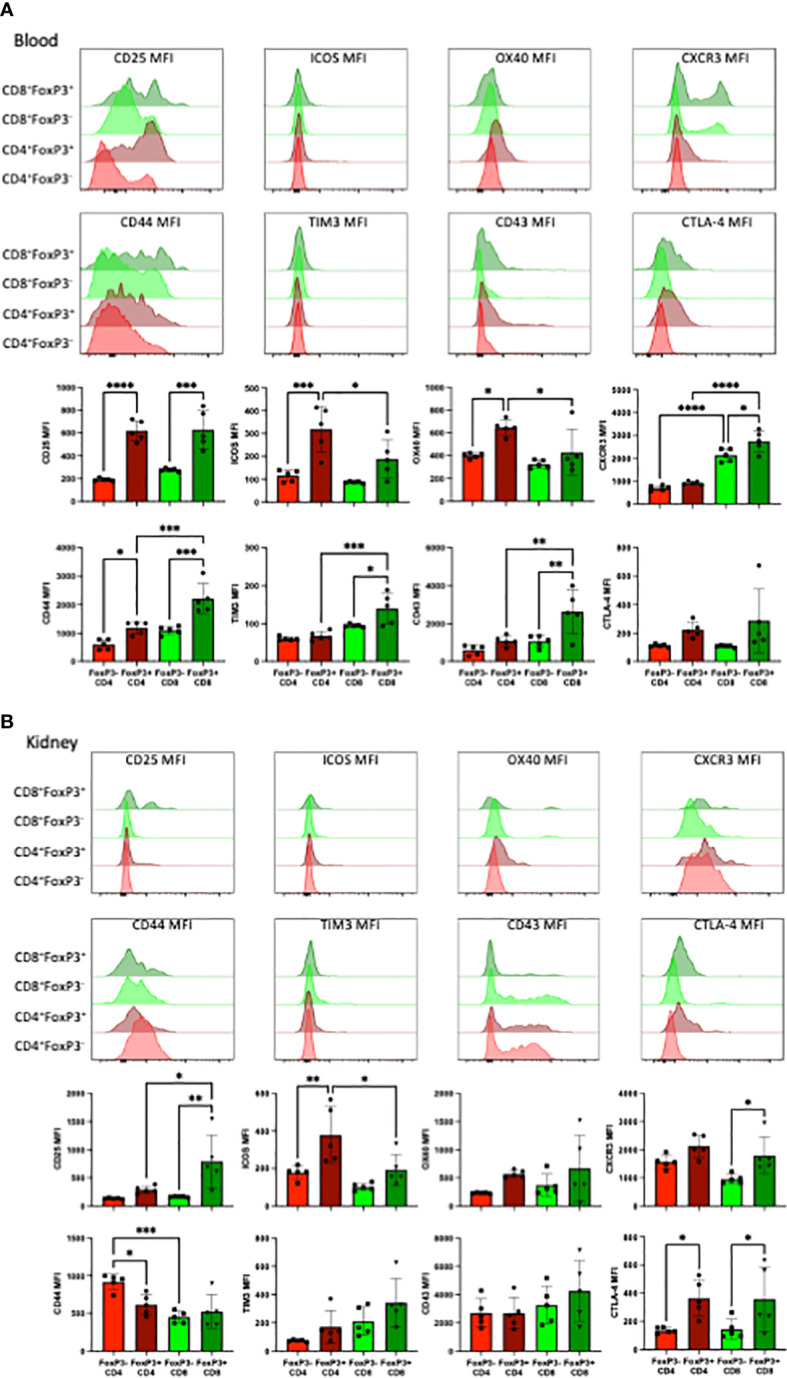
FoxP3^+^CD8^+^ T cells exhibit distinct cell surface expression profiles compared to FoxP3^+^CD4^+^ T cells in the blood and kidney. **(A)** Representative flow cytometric data of the indicated cell surface proteins expressed on total CD4^+^ and CD8^+^ T cells in the blood stratified by FoxP3 expression. **(B)** Representative flow cytometric data of the indicated cell surface proteins expressed on tissue resident CD4^+^ and CD8^+^ T cells in the kidney stratified by FoxP3 expression. Experiment shown is representative of 2 independent experiment with a total of 8-10 mice per group. *p < 0.05, **p < 0.01, ***p < 0.001, ****P < 0.0001 by one way ANOVA correcting for multiple comparisons.

The phenotype of FoxP3^+^ T cells in groups that received TCD and received anti-CD28dAb were further explored by comparing the expression of several cell surface markers on FoxP3^-^ versus FoxP3^+^ cells within both the CD4^+^ and CD8^+^ T cell compartments (**Figures 5A, B**). In the blood, CD4^+^ and CD8^+^ FoxP3^+^ T cells expressed higher levels of CD25, ICOS, and OX40 compared with CD4^+^ and CD8^+^ FoxP3^-^ T cells. Additionally, CXCR3, CD44, TIM3, and CD43 were more highly expressed on CD8^+^FoxP3^+^ T cells than on CD8^+^FoxP3^-^ T cells or on CD4^+^FoxP3^+^ T cells (**Figure 5A**). In kidney resident cells, CD25 was more highly expressed on CD8^+^FoxP3^+^ T cells compared to CD8^+^FoxP3^-^ and CD4^+^FoxP3^+^ T cells. ICOS was elevated on CD4^+^FoxP3^+^ and CD8^+^FoxP3^+^ T cells compared to CD4^+^FoxP3^-^ and CD8^+^FoxP3^-^ T cells. CXCR3 was elevated on CD8^+^ FoxP3^+^ T cells compared to CD8^+^FoxP3^-^ T cells (p=0.020). CD44 expression was decreased on CD4^+^FoxP3^+^ T cells compared to CD4^+^FoxP3^-^ T cells (p=0.020) and was further decreased on CD8^+^ FoxP3^-^ T cells compared to CD4^+^FoxP3^-^ T cells (p=0.001). There was no difference in the expression of OX40, TIM3, or CD43 on FoxP3^-^ vs FoxP3^+^ T cells in the kidney. CTLA-4 was elevated on CD4^+^ FoxP3^+^ and CD8^+^ FoxP3^+^ T cells compared to CD4^+^ FoxP3^-^ and CD8^+^ FoxP3^-^ T cells. (**Figure 5B**).

## Discussion

The aim of this study was to understand the effect of CD28 blockade on the immune system during TCD and immune reconstitution in the setting of transplantation. The role of CD28 on T cell homeostatic proliferation has been investigated in the past by Prlic et al. Using CD28 knockoout mice, their group revealed that CD28 costimulation was dispensible for homeostatic expansion of CD4^+^ and CD8^+^ T cells (28). Results from our study are largely consistant with this finding, though we did observe a small impact of CD28 blockade on the early kinetics of CD4 T cell reconstitution which may be due to the fact that we tracked the absolute count of T cells rather than the number of divisions (**Figure 1D**). It has also been established that TCD induces a transient memory-like phenotype in naïve T cells, marked by upregulation of CD44 (46–48). Our study reveals that CD28 blockade prevents homeostatically proliferated T cells from acquring this CD44^+^ memory phenotype, which might help to prevent allorejection (49).

ATG induction therapy is routinely used in the clinic to deplete donor-reactive immune cells. While in this study we utilize antibodies directed against CD4^+^ and CD8^+^ T cells to investigate the effect of TCD and homeostatic reconstitution, ATG is known to have more widespread effects on the immune system. Beyond TCD, ATG induces the apoptosis of B cells, modulates surface molecules that mediate leukocyte interactions, and induces the expansion of Tregs *ex vivo* (50). In mice, naïve T cells are more susceptible to ATG-mediated depletion than memory and effector T cells, with CD4^+^ T cells being more resistant to depletion than CD8^+^ T cells (51, 52). Therefore, it is possible that ATG induction therapy could influence the immune landscape during CD28 blockade in a different manner than anti-CD4/anti-CD8-mediated depletion.

It has been well established that CD4^+^FoxP3^+^ Tregs require CD28 signaling for thymic development and homeostatic maintenance. While we found that the frequency of CD4^+^FoxP3^+^ Tregs was reduced in the anti-CD28dAb treated groups, it has been previously demonstrated that these Tregs are still able to effectively function through preserved CTLA-4 signaling (14, 53). We show that in the kidney, CD4^+^ and CD8^+^ FoxP3^+^ T cells express greater amounts of CTLA-4 than CD4^+^ and CD8^+^ FoxP3^-^ T cells, so it may follow that they are also governed by CD28 signaling. Nonobese diabetic (NOD) mice that lack CD28 signaling are deficient in CD4^+^ Tregs which results in exacerbated onset of spontaneous diabetes (54). Tang et al. (55) demonstrated that CD28 signaling affects CD4^+^ Treg homeostasis both directly by upregulating CD25 expression on these cells and indirectly by inducing IL-2 production by conventional T cells, which in turn provides survival signals to Tregs. Furthermore, studies utilizing CTLA-4-Ig (abatacept) to treat type 1 diabetes and rheumatoid arthritis have shown a reduction in the frequency of CD4^+^ Tregs in these patients (56, 57). Our findings in the CD4^+^Foxp3^+^ Treg compartment are consistent with these results. However, the requirement for CD28 signaling on CD8^+^Foxp3^+^ T cells has not been well studied, and our results suggest that in contrast to CD4^+^ Tregs, CD8^+^ Foxp3^+^ T cells exhibit a reduced requirement for CD28 signaling and are therefore not negatively impacted by CD28 blockade *in vivo* in the setting of transplantation.

Not only did we find that CD28 blockade did not inhibit the survival of CD8^+^ Tregs, instead, in the context of TCD, CD28 blockade actually promoted the expansion or accumulation of this population. Previous studies using RNA-silencing experiments have shown that CD8^+^Foxp3^+^ T cells exhibit suppressive function; specifically that FOXP3-knock down on CD8^+^ T cells significantly reduces their ability to suppress both CD4^+^ T cell proliferation and the production of autoantibodies (58). Moreover, previous reports have shown that CD8^+^FoxP3^+^ T cells express CD25, CTLA-4, and ICOS (43, 59–62), findings which are supported by our data in the context of TCD and transplantation. These surface receptors have been shown to contribute to the functional suppressive activity of both CD4^+^ and CD8^+^ regulatory T cells (43, 59, 63–65). For example, one study found that CD8^+^FoxP3^+^ T cells inhibit the upregulation of costimulatory molecules on dendritic cells, suppress CD4^+^ and CD8^+^ T cell proliferation, and, following adoptive transfer, protect full MHC mismatched skin allografts from rejection (43). Moreover, CD8^+^FoxP3^+^ T cells have been shown to be induced to differentiate from CD8^+^FoxP3^-^ T cells *via* exposure to TGF-β and TCR stimulation (61). While CD8^+^FoxP3^+^ cells are typically present at frequencies much lower than their CD4^+^FoxP3^+^ counterparts, a previous study reported that frequencies of CD8^+^FoxP3^+^ T cells were increased in RA patients treated with T cell depletional therapy (59), a finding which is in line with our results. Whether the increased frequency of CD8^+^FoxP3^+^ T cells that we observe in the blood and kidney during CD28 blockade following TCD and homeostatic reconstitution are induced pharmacologically or are naturally occurring, and the specific suppressive capacity of these cells in this setting, remains to be elucidated.

One population of T cells that may be differently impacted by TCD during selective CD28 blockade is T_RM_. These cells are characterized by their expression of CD69 and CD103 (30, 31), and it has been shown that lymphopenia-induced naïve T cells can traffic to tissues and differentiate into memory T cells (46, 66). T_RM_ have been identified as key mediators of allograft rejection (52) and it has been shown in a murine kidney transplant model that treatment with cyclosporine does not prevent CD8^+^ T cells from acquiring a T_RM_ phenotype (29). In human transplant nephrectomies, both donor and recipient derived T_RM_ have been found in transplanted kidneys, with donor derived T_RM_ found in early acutely rejected allografts and recipient derived T_RM_ found in later chronically rejected kidney allografts (67). Another study found the PD-1/PD-L1 pathway to be important for regulating the effector function of T_RM_ in the human pancreas (68). In the kidney we found that anti-CD28dAb alone reduced the frequency of resident PD-1^+^ CD4^+^ and CD8^+^ T cells compared to no treatment, but not following homeostatic reconstitution. This preserved PD-1 expression suggests that kidney resident CD4^+^ and CD8^+^ T cells may be more susceptible to immune regulation *via* PD-L1 during TCD+CD28dAb. While we observed that selective CD28 blockade alone reduced the frequency of CD4^+^ and CD8^+^ T_RM_ in the kidney compared to no treatment, the fact that this was not seen following T cell reconstitution may indicate a reduced susceptibility of this compartment to the effects of CD28dAb.

Taken together, these data support the utilization of combined TCD and selective CD28 blockade to prevent allograft rejection. The emergence of a population of CD8^+^FoxP3^+^ T cells with a cell surface expression profile distinct from CD4^+^FoxP3^+^ T cells might have implications in transplantation and warrants further investigation.

## Data availability statement

The raw data supporting the conclusions of this article will be made available by the authors, without undue reservation.

## Ethics statement

The animal study was reviewed and approved by Emory University Institutional Animal Care and Use Committee (IACUC).

## Author contributions

MF conceived of the study and obtained funding. JH, DL, RC, and MW performed experiments and collected data. JH, DL, RC, and MF analyzed and interpreted data. JH and MF wrote the paper. All authors contributed to the article and approved the submitted version.
